# The Wnt/β-Catenin Signaling Pathway Tips the Balance Between Apoptosis and Reprograming of Cell Fusion Hybrids

**DOI:** 10.1002/stem.515

**Published:** 2010-11

**Authors:** Frederic Lluis, Elisa Pedone, Stefano Pepe, Maria Pia Cosma

**Affiliations:** a Telethon Institute of Genetics and Medicine (TIGEM)CNRNaples, Italy; b Institute of Genetics and Biophysics (IGB)CNR, Naples, Italy; c Center for Genomic Regulation (CRG)Barcelona, Spain; d Institució Catalana de Recerca i Estudis Avançats (ICREA)Barcelona, Spain

**Keywords:** cell reprogramming, Wnt/beta-catenin pathway, spontaneous cell-cell fusion

## Abstract

Cell-cell fusion contributes to cell differentiation and developmental processes. We have previously showed that activation of Wnt/β-catenin enhances somatic cell reprograming after polyethylene glycol (PEG)-mediated fusion. Here, we show that neural stem cells and ESCs can fuse spontaneously in cocultures, although with very low efficiency (about 2%), as the hybrids undergo apoptosis. In contrast, when Wnt/β-catenin signaling is activated in ESCs and leads to accumulation of low amounts of β-catenin in the nucleus, activated ESCs can reprogram somatic cells with very high efficiency after spontaneous fusion. Furthermore, we also show that different levels of β-catenin accumulation in the ESC nuclei can modulate cell proliferation, although in our experimental setting, cell proliferation does not modulate the reprograming efficiency per se. Overall, the present study provides evidence that spontaneous fusion occurs, while the survival of the reprogramed clones is strictly dependent on induction of a Wnt-mediated reprograming pathway. STEM CELLS 2010;28:1940–1949

## INTRODUCTION

Cell-cell fusion regulates many developmental processes. Fertilization, muscle and bone development, and syncytiotrophoblast formation are all known examples of cell-cell fusions [1]. However, cell-cell fusion might also regulate cell fate and cell differentiation. Neural stem cells (NSCs) and bone marrow cells (BMCs) have indeed been shown to fuse spontanously with embryonic stem cells (ESCs), even if this occurs at a very low efficiency [2,3]. The few hybrid clones selected were shown to have a stem cell-like phenotype. The stem cell features of ESCs were dominant over the somatic cell traits and allowed the reprograming of the somatic cell nucleus. Thus, cell-cell fusion is a way to force the fate of a cell, and in the case of fusion with ESCs, this mechanism induces cellular reprograming, that is, dedifferentiation of somatic cells [4].

We have recently shown that fusion-mediated reprograming of a somatic cell is greatly enhanced by time-dependent activation of the Wnt/β-catenin signaling pathway. After Wnt binding to its receptors or inhibition of glycogen synthase kinase-3 (GSK3), as a component of the destruction complex, β-catenin is stabilized and translocates into the nucleus, where it activates several target genes. ESCs treated for 24 hours with Wnt3a or with the GSK3 inhibitor, 6-bromoindirubin-3′-oxime (BIO), can reprogram somatic cells after polyethylene glycol (PEG)-mediated fusion [5].

In vivo cell-cell fusion has also been seen after bone marrow (BM) transplantation. BM-derived cells fuse at low efficiency with hepatocytes, kidney cells [6,7], muscle cells [8,9], and even Purkinje cells in the cerebellum [10–12]. Chronic inflammation increases the fusion efficiency in the brain. However, even if in vivo fusion has been described in many reports, its efficiency appears to be extremely low, which has also resulted in some skepticism around the physiology of some studies [4]. It should also be noted that if cell-cell fusion is a mechanism that regulates cell fate and cell differentiation, then this mechanism must be finely tuned; potentially, the destiny of the majority of hybrids that are spontaneously formed is to undergo cell death via apoptosis. Indeed, ESCs have an apoptotic machinery and an antiapoptotic defense program. When subjected to prolonged hypoxia or oxidative stress induced with H_2_O_2_ or citrinin, they embark on apoptosis [13–15].

Here, we show that ESCs fuse with NSCs spontaneously at low efficiency (about 2%) and that the resulting fusion hybrids will undergo apoptosis unless the Wnt/β-catenin pathway is activated, whereupon they undergo reprograming and cell proliferation. Only a low level of β-catenin in the nucleus of ESCs is necessary for these cells to reprogram somatic cells after their spontaneous fusion. Our data demonstrate that the Wnt/β-catenin signaling pathway is the cell-fate switch that promotes reprograming of spontaneous hybrids against their apoptosis-mediated cell death.

## MATERIALS AND METHODS

### Cells

Neural stem (NS)-Oct4-puro cells were isolated from HP165 mice and they carry the regulatory sequences of the mouse *Oct4* gene driving green fluorescent protein (GFP) and puromycin-resistance genes. The NS-Oct4-puro cells were a gift from Dr. A. Smith (Wellcome Trust Centre for Stem Cell Research, University of Cambridge, Cambridge, U.K.) and were cultured as previously described [16]. Hygromycin-resistant mouse embryonic fibroblasts (MEFs) were purchased at passage three (Millipore, Billerica, MA, USA, http://www.millipore.com). Embryonic stem (ES)-neo cells were derived from E14Tg2a and transduced with the lentiviral pHRcPPT-PGK-Neomycin vector. ESCs were cultured on gelatin in knockout Dulbecco's modified Eagle's medium supplemented with 20% fetal bovine serum (Hyclone, South Logan, UT, USA, http://www.thermoscientific.com), 1× nonessential amino acids, 1× GlutaMax, 1× 2-mercaptoethanol, and 1,000 U/ml leukemia inhibitory factor (LIF) ESGRO (Chemicon, Billerica, MA, USA, http://www.millipore.com).

### Cell Hybrids

ESC + NSC and ESC + MEF cocultures: 1 × 10^6^ ESCs were plated onto preplated 1 × 10^6^ NSCs or MEFs, first for 2 hours in NSC or MEF medium, respectively, and then for 2 hours in ESC medium. The cells were then trypsinized, and plated at 20% into gelatin+laminin-treated p100 dishes in ESC medium without or with 1 μM BIO (Calbiochem, Darmstadt, Germany, http://www.emdchemicals.com/life-science-research/calbiochem), for different times. After 72 hours, puromycin or hygromycin plus neomycin were added to the ES medium for hybrid selection.

For cell treatment with carbobenzoxy-valyl-alanyl-aspartyl-[O-methyl]- fluoromethylketone (z-VAD)-FMK (R&D Systems, Minneapolis, MN, USA, http://www.rndsystems.com/) the inhibitor was dissolved in dimethylsulphoxide and incubated with the cells (20 μM) for 36 hours. Seventy-two hours after coculture, puromycin was added to the ES medium for hybrid selection.

### Plasmid Construction and Stable ES Clone Generation

Mouse β-catenin mutated at serine 33 was a gift from Dr. de la Luna (Centre for Genomic Regulation, Barcelona, Spain) and it was subcloned into the empty pCAG-C1 vector, which contained, in sequence order: CAG promoter, a multicloning site, internal ribosome entry site, neomycin-resistance gene, and polyA. Stable ESC lines expressing β-catenin were isolated after nucleofection (Amaxa, Basel, Switzerland, http://lonza.com/group/en/company.html) of this construct, and drug selection was performed with 250 μg/ml neomycin, as previously described.

### Transient Transfections and Luciferase Activity

ESCs were cotransfected by nucleofection (Amaxa) with the Topflash reporter construct driving firefly luciferase cDNA [5] and pRL-CMV driving constitutive expression of *Renilla* cDNA for normalization. The cells were lysed with 1× passive reporter lysis buffer. The firefly and *Renilla* reporter activities were measured using a 96-well-based luminometer, with detection according to manufacturer instructions (Promega Dual-Light system, Madison, WI, USA, http://www.promega.com).

### Western Blotting

Western blotting was performed as previously described [17]. The primary antibodies used were: anti-phospho-c-Myc (Thr58/Ser62; #9401 Cell Signaling Technologies, Danvers, MA, USA, http://www.cellsignal.com/); anti-c-Myc (N-262) sc-764 (Santa Cruz Biotechnology, Santa Cruz, CA, USA, http://www.scbt.com/); and anti-β-tubulin, clone D66, T0198 (Sigma-Aldich, St. Louis, MO, USA, http://www.sigmaaldrich.com/).

### Semiquantitative RT-PCR Analysis

For reverse transcriptase PCR (RT-PCR), total RNA was extracted from ESCs and from embryoid bodies (200 embryoid bodies for each clone and for each differentiation time point) using RNeasy kits (Qiagen, Washington, DC, USA, http://www.qiagen.com/), and the cDNA was generated using superscript III (Invitrogen, Carlsbad, CA, USA, http://www.invitrogen.com/site/us/en/home.html). The primers used were:

*Oct4*: forward, GGCGTTCTCTTTGGAAAGGTGTTC; reverse, CTCGAACCACATCCTTCTCT;

*Nanog*: forward, AGGGTCTGCTACTGAGATGCTCTG; reverse, CAACCACTGGTTTTTCTGCCACCG;

*Rex1*: forward, GCCCTCGACAGACTGACCCTAA; reverse, CTTCCTCAGGGCGGTTTTACCC;

*Fgf4*: forward, GACTACCTGCTGGGCCTCAA; reverse, CGACACTCGGTTCCCCTTCT;

*Olig2*: forward, GCGTGGGTATCAGAAGCACT; reverse, CCAGTCGGGTAAGAAACCAA;

*Blbp*: forward, GGGTAAGACCCGAGTTCCTC; reverse, ATCACCACTTTGCCACCTTC;

*Brachyury*: forward, TGCTGCCTGTGAGTCATA; reverse, ACAAGAGGCTGTAGAACATG;

*Nkx2.5*: forward, GCTCTCCTGCTTTCCCAGC; reverse, CTCCCATCCCTACTGCCTTCTGCAGC;

Alpha-fetoprotein (*AFP*): forward, TCCCTCATCCTCCTGCTA; reverse, GCACAT TCTTCTCCGTCAC;

glyceraldehyde 3-phosphate dehydrogenase (*GAPDH*): forward, ACTCCCACTCTTCCACCTTC; reverse, TCTTGCTCAGTGTCCTTGC.

### Bisulphite Genomic Sequencing

Bisulphite treatment was performed using Epitect Bisulphite kits (Qiagen), according to the manufacturer recommendations. The amplified products were cloned into pCR2.1-TOPO (Invitrogen). Ten randomly selected clones were sequenced with the M13 forward and M13 reverse primers for each gene, as follows:

MeNanog: forward, GATTTTGTAGGTGGGATTAATTGTGAATTT; reverse, ACCAAAAAAACCCACACTCATATCAA TATA;

MeOct4: forward, GGTTTTTTAGAGGATGGTTGAGTG; reverse, TCCAACCCTACTAACCCATCACC.

### FACS Analysis

Twenty-four hours before fusion, the NS-Oct4-puro cells were stained with Vybrant DiO (5 μl/ml; Invitrogen) in NSC medium for 20 minutes at 37°C. The cells were thoroughly rinsed (three times) with phosphate-buffered saline (PBS) before being trypsinized and replated in t25 flasks. The day after, the untreated and treated ESCs (BIO or Wnt3a, up to 48 hours) were stained with Vybrant DiD (5 μl/ml; Invitrogen) in ESC medium for 20 minutes at 37°C, washed, trypsinized, counted, and plated on NS-Oct4-puro cells. After 4 hours of coculture, the total cells were trypsinized and divided: 80% were used for fluorescence activated cell sorting (FACS) analysis and 20% were plated for reprograming analysis. The cells used for FACS analysis were centrifuged and resuspended in PBS with 0.1% bovine serum. FACS analyses were performed using a BD Biosciences FACSAria cytometer (Franklin Lakes, New Jersey, USA, http://www.bdbiosciences.com/home.jsp).

### In Vitro Differentiation of Reprogramed Cells

The differentiation medium for the production of embryoid bodies consisted of ES medium without LIF. The cells were harvested by trypsinization, counted, and propagated in hanging drops (400 single ES cells/30 μl initial drop) for 2 days, before being transferred to 10-cm^2^-bacterial dishes. On day 5, the embryoid bodies were transferred onto gelatinized p100 dishes.

### Teratoma Production

Cells were trypsinized into single-cell suspensions and resuspended in PBS to a concentration of 1.5 × 10^7^ cells/ml. These cells were injected subcutaneously into the hind limbs of Fox Chase severe combined immunodeficiency (SCID) mice (in 200 μl) using a 25-gauge needle. Teratomas were collected after 4 weeks, and they were fixed, embedded, sectioned, and H&E stained.

### Quantitative RT-PCR

RNA isolation and reverse transcription were carried out as described for semiquantitative RT-PCR. The template for each PCR reaction was the cDNA obtained from 16 ng total RNA in a 25-μl reaction volume. Platinum SYBR green qPCix-UDG (Invitrogen) was used with an ABprism 7000 real-time PCR machine, according to the manufacturer recommendations. The primers used were:

p16RT: forward, GTGTGCATGACGTGCGGG; reverse, GCAGTTCGAATCTGCACCGTAG; p19RT: forward, GCTCTGGCTTTCGTGAACATG; reverse, TCGAATCTGCACGCTAGTTGAG.

### Cell Proliferation Analysis Using 5-(and 6-) Carboxyfluorescein Diacetate Succinimidyl Ester Staining

ES cells were labeled with 5-(and 6-)carboxyfluorescein diacetate succinimidyl ester (CFSE; Molecular Probes, Carlsbad, CA, USA, http://www.invitrogen.com/site/us/en/home.html) at a concentration of 5 μM, for 15 minutes in PBS with 1% BSA at 37°C. The cells were washed several times with serum-containing medium, and then tested for cell viability using Trypan blue exclusion. Following culture for 12–24 hours with complete medium, flow cytometry was performed with a FACS Aria (BD Biosciences) and CFSE was analyzed in Modfit software.

### Cell Proliferation Analysis Using 5-bromo-2-deoxyuridine (BrdU) Assay

Cells (1 × 10^4^) were treated with or without purified Wnt3a (100 ng/ml) or BIO (1 μM) up to 48 hours. Quantitation of BrdU positive cells (3 hours of BrdU treatment), which indicates the number of cells that enter S-phase, was through the BrdU Cell Proliferation Assay (Chemicon), according to the manufacturer protocol.

### Cell Viability Assay

Cells (1 × 10^4^) were treated with or without H_2_O_2_ (250 μM) and citrinin (60 μM) at 37°C for 24 hours. Quantitation of ATP, which indicates the presence of metabolically active cells, was through the CellTiter-Glo Luminiscent Cell Viability Assay (Promega), according to the manufacturer protocol.

### Cell Apoptosis Assay

Cells (1 × 10^4^) were treated without or with H_2_O_2_ (250 μM) and citrinin (60 μM) at 37°C for 24 hours. Oligonucleosomal DNA fragmentations (a hallmark of apoptosis) were measured using Cell Death Detection ELISA^plus^ kits, according to the manufacturer protocol (Roche Molecular Biochemicals, Basel, Switzerland, http://www.roche.com/index.htm). Spectrophotometric data were obtained at 405 nm using an ELISA reader.

## RESULTS

### Activation of Wnt/β-Catenin Pathway Does Not Protect ESCs from Entering Apoptosis

**Figure 1:**
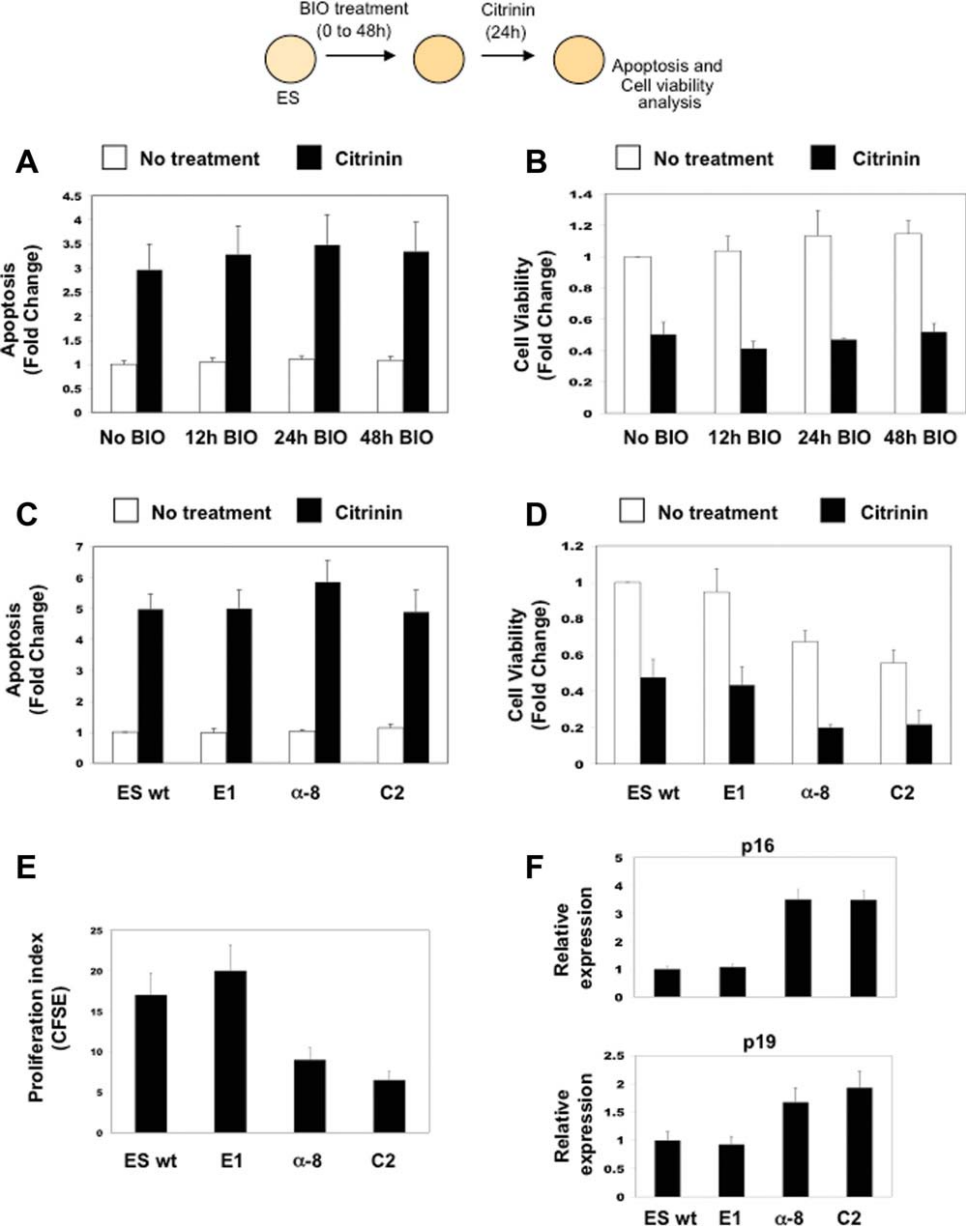
Effects of citrinin in BIO-treated ESCs and ES-β-catenin clones. **(A, B):** ES cells (1 × 10^4^ cells) without and with BIO treatment for the indicated times and **(C, D)** ES β-catenin clones. To evaluate the effects of β-catenin accumulation in the control of apoptosis, ES BIO-treated cells and ES β-catenin clones were then treated with citrinin (60 μM) for 24 hours. **(A, C):** Apoptosis was evaluated using Cell Death Detection Elisa kits (mean ± SEM; *n* = 3). **(B, D):** Cell viability was determined by quantitation of ATP with the Cell Titer-Glo Luminescent Cell Viability Assay (mean ± SEM; *n* = 3). Fold changes were calculated with respect to No BIO samples. **(E):** ES β-catenin clones were analyzed for proliferation potential using the FACS-CFSE system (mean ± SEM; *n* = 3). **(F):** Quantitative RT-PCR analysis of *p16* and *p19* transcript levels in the ES-β-catenin clones (mean ± SEM; *n* = 3). Abbreviations: BIO, 6-bromoindirubin-3′-oxime; CFSE, 5-(and 6-) carboxyfluorescein diacetate succinimidyl ester; ES, embryonic stem; FACS, fluorescence activated cell sorting; RT-PCR, real time PCR; wt, wild type.

Activation of Wnt/β-catenin signaling controls ESC self-renewal and enhances somatic cell reprograming [5,18,19]. Wnt signaling can be activated by the inhibition of GSK3 with BIO [20], which results in the nuclear accumulation of β-catenin. Low levels of nuclear β-catenin accumulation can be obtained by culturing ESCs in 1 μM BIO-containing medium [5]. The ESCs were thus cultured in BIO for 12, 24, and 48 hours, and then harvested to analyze their viability and apoptotic phenotype. The different BIO treatments did not modify the cell viability or promote a decrease or increase in apoptosis over the untreated ESCs (Fig. 1A, 1B; compare white bars). In contrast, ESCs treated with 250 μM H_2_O_2_ or with 60 μM citrinin (Fig. 1A, 1B; compare No BIO white and black bars and data not shown) showed increased apoptosis and decreased viability, as previously shown [13–15]. Interestingly, when the ESCs were precultured with BIO for different times and then treated with H_2_O_2_ or citrinin, BIO did not protect the cells from apoptosis or increase their viability, ruling out a role for Wnt signaling in an anti-apoptotic pathway (Fig. 1A, 1B and data not shown).

To confirm that the Wnt canonical pathway did not activate transcription of antiapoptotic genes or of genes enhancing cell survival, we analyzed a variety of ES clones expressing different levels of β-catenin [5]. The activity of β-catenin in the different clones was evaluated by testing the expression of the reporter FOPflash and TOPflash genes (luciferase gene under the control of the transcription factor/lymphoid enhancer factor (TCF/LEF)-containing promoter [18]; supporting information Fig. S1). Although the clones expressed different levels of β-catenin, they showed comparable levels of apoptosis, with respect to wild-type ESCs. This held true for both untreated and H_2_O_2_/ citrinin-treated cells (Fig. 1C and data not shown). The cell viability of all of the β-catenin ESC clones or of the wild type (WT) cells treated with H_2_O_2_ or citrinin decreased with respect to the untreated clones (Fig. 1C, 1D and data not shown). Thus, both high and low levels of nuclear β-catenin accumulation did not protect the cells from apoptosis or increase their survival. Of note, there was a decrease in cell viability in the untreated clones expressing medium or high levels of β-catenin (the α-8 and C-2 clones; Fig. 1D; compare white bars). This was probably due to reduced cell proliferation (measured by the CFSE assay) and to upregulation of the cyclin inhibitor genes *p16^Ink4a^* and *p19^Arf^* (Fig. 1E, 1F).

All in all, these data show that in ESCs, the Wnt/β-catenin pathway does not regulate and/or modulate either the apoptotic machinery or cell viability.

### Activation of the Wnt/β-Catenin Pathway Enhances Reprograming of Hybrids Spontaneously Formed Between ESCs and Somatic Cells

ESCs were shown to fuse spontaneously (without PEG) with NSCs and BMCs, although with a very low efficiency that gives rise to few reprogramed clones [2,3]. Thus, we hypothesized that the majority of hybrids did not undergo reprograming but instead underwent apoptosis. Here, we investigated whether the activation of the Wnt/β-catenin signaling pathway enhances reprograming of spontaneously fused cells, with the consequence that a high number of clones can be selected.

**Figure 2:**
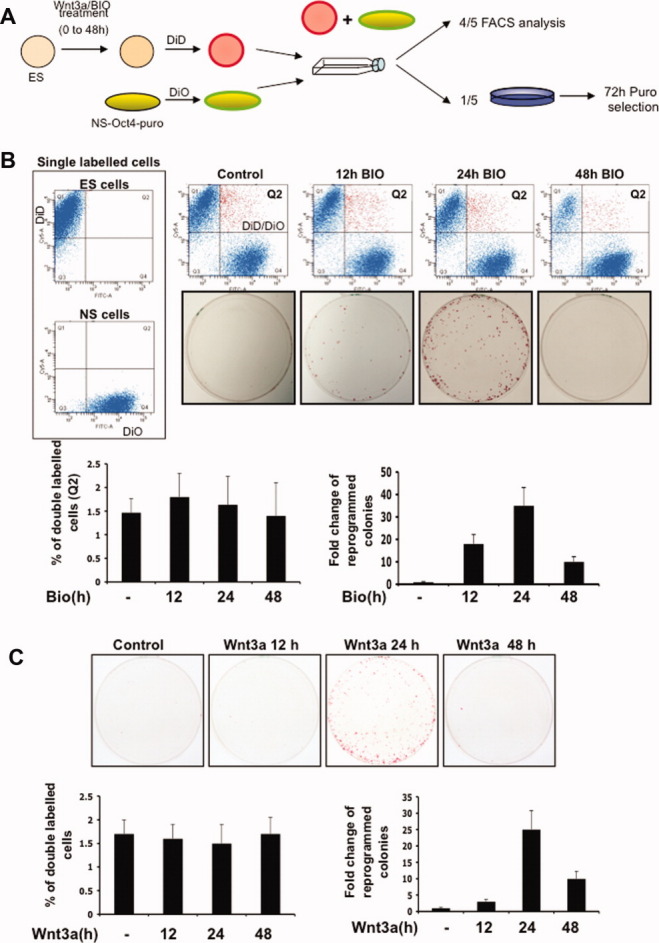
Activation of the Wnt/β-catenin pathway increases reprograming but not spontaneous cell fusion. **(A):** Schematic representation of the experimental plan. ESCs without and with treatment with Wnt3a or BIO were DiD stained and cocultured with DiO-stained NS-Oct4-puro cells. The cells were then divided for FACS or reprograming determinations. **(B):** ES cells pretreated with BIO. Quantification of FACS analysis (% total double-labeled cells) and representative growth plates with quantification of reprograming efficiency (fold-increase in colony number) of the cocultured cells (mean ± SEM; *n* = 3). **(C):** Quantification of FACS analysis and reprograming efficiency, with representative growth plates, when ES cells were preatreated with Wnt3a (mean ± SEM; *n* = 3). Abbreviations: BIO, 6-bromoindirubin-3′-oxime; ES, embryonic stem; FACS, fluorescence activated cell sorting; NS, neural stem.

We then evaluated whether the pretreatment of ESCs with BIO or Wnt3a, and their subsequent coculture with NS-Oct4-puro/GFP cells (called NS-Oct4-puro cells) allowed the reprograming of spontaneously fused cells. NS-Oct4-puro cells expressed the *PuroR* gene and GFP under the control of the *Oct4* promoter, which is only active in pluripotent cells. ESCs were incubated with the cell membrane dye DiD (red dye), pretreated with 1 μM BIO or 100 ng/ml Wnt3a for 12, 24, and 48 hours, and then seeded into dishes where DiO (green)-labeled NS-Oct4-puro cells had previously been plated. These cells were cocultured for 4 hours, to allow the formation of fusion hybrids. The hybrids were then trypsinized and the cell suspension was divided into two parts. Eighty percentage of the cells were analyzed by FACS, and the remaining 20% were replated, to select for the reprogramed clones. The hybrids were selected in ESC medium supplemented with puromycin (Fig. 2A). Under these culture conditions, only reprogramed hybrids can survive, proliferate, and grow [5,21]. The resistant colonies were stained for the expression of alkaline phosphatase (AP), an ESC marker, and counted. These cells had been reprogramed, as they retained a rounded ESC-like phenotype (not shown) and expressed AP and Oct4-puro/GFP. We observed a high number of reprogramed clones, with up to a 45-fold increase (400 reprogramed clones on average) after 24 hours of BIO culturing, and up to a 35-fold increase (300 reprogramed clones on average) after 24 hours of Wnt3a treatment (Fig. 2B, 2C). The number of reprogramed clones decreased after BIO treatments of 48 hours, indicating that prolonged BIO culturing reduces reprograming efficiency, as we have previously shown [5]. These data clearly show that time-dependent activation of the Wnt/β-catenin pathway in ESCs allows these cells to reprogram NSCs after their spontaneous fusion.

BIO and Wnt3a did not enhance fusion, as the same percentages of fused cells (Q2 fraction, double-dyed DiD/DiO cells) were counted by FACS analysis following coculturing of the hybrids formed in the absence and presence of BIO or Wnt3a for the different times (Fig. 2B, 2C). Only 2% of the cells fused spontaneously, and in the absence of reprograming, very few clones could be selected. In contrast, when the Wnt/β-catenin signaling pathway was activated for a specific time (24 hours of treatment), reprograming was strongly enhanced, and the majority of the hybrids were then selected and propagated as pluripotent clones with self-renewal ability.

In addition, the Wnt3a and BIO treatments did not increase the proliferation rate of ESCs, as measured in BrdU-positive ESCs (supporting information Fig. S2). This rules out that the increased reprograming seen was dependent on a Wnt3a or BIO-dependent increase in proliferation.

Interestingly, we also observed the reprograming of hybrids when we first allowed hybrids to form by coculturing ESCs either with NS-Oct4-puro cells or with MEFs, and subsequently activated the Wnt pathway with BIO. After 24 hours of BIO culturing of the hybrids, there was up to a 45-fold reprograming increase for fusion between ESCs and NSCs (650 reprogramed clones on average) and up to an eightfold increase for fusion between ESCs and MEFs (450 reprogramed clones on average), as compared with the fusions with untreated ESCs (supporting information Fig. S3).

**Figure 3:**
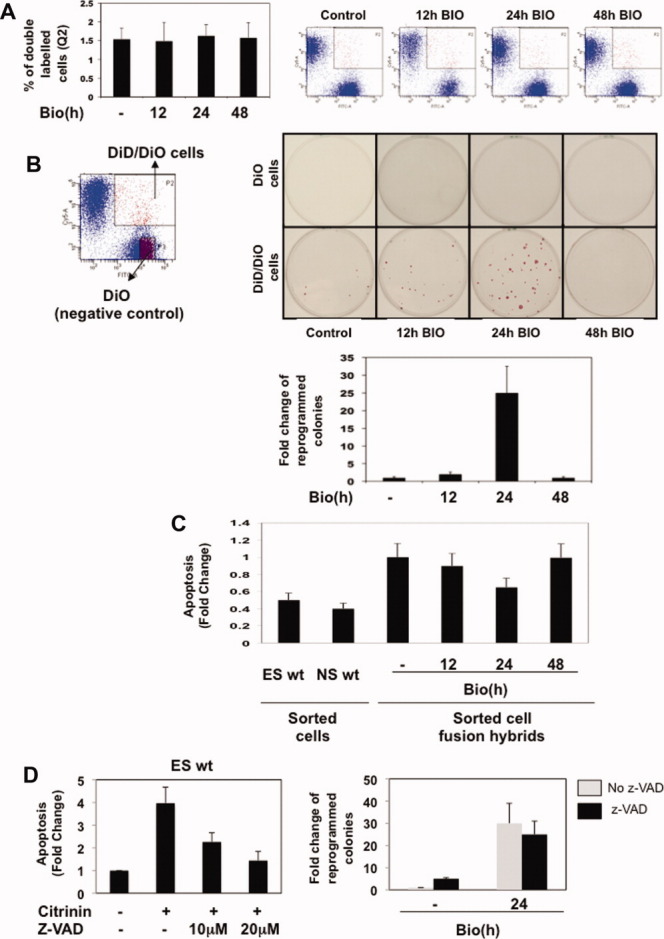
Analysis of reprograming, fusion efficiency, and apoptosis of FACS-sorted hybrids. FACS analysis and reprograming efficiency, and their quantification for spontaneous fusion of BIO-pretreated ESCs (DiD labeled) with NS-Oct4-puro cells (DiO labeled). **(A):** The cells were cocultured (1 × 10^7^ cells/plate) for 4 hours (2 hours with NS medium, plus 2 hours with ES medium), trypsinized, and the hybrids were analyzed by FACS, as % double-labeled cells (histogram; mean ± SEM; *n* = 4), with representative FACS analyses also shown. **(B):** Eighty thousand single-labeled (green) or double-labeled (red+green) FACS-sorted cells were replated in p100 plates. The clones were puromycin selected, stained for the expression of alkaline phosphatase, and counted. Quantification of the fold change increases in the reprogramed colonies is also shown (mean ± SEM; *n* = 4), along with representative FACS analysis and growth plates. **(C):** 1 × 10^4^ sorted ESCs and NSCs as well as 1 × 10^4^ sorted cell fusion hybrids were plated and apoptosis was evaluated using Cell Death Detection Elisa kits 48 hours after sorting (mean ± SEM; *n* = 3). Fold changes were calculated with respect to No BIO cell fusion hybrids. **(D):** ES cells (1 × 10^4^ cells) were treated with citrinin (60 μM) and with or without z-VAD for 24 hours (Left panel). Apoptosis was evaluated using Cell Death Detection Elisa kits (mean ± SEM; *n* = 3). Quantification of reprograming efficiency (fold-increase in colony number) of the cocultured cells between untreated or 24 hours-BIO-treated ES cells with NS cells (right panel). After that, cocultured cells were trypsinized, plated, and treated or untreated for 36 hours with z-VAD (20 μM; mean ± SEM; *n* = 3). Abbreviations: BIO, 6-bromoindirubin-3′-oxime; ES, embryonic stem; FITC, fluorescein isothiocyanate; NS, neural stem; wt, wild type; z-VAD, carbobenzoxy-valyl-alanyl-aspartyl-[O-methyl]- fluoromethylketone.

To further confirm these results, we sorted ES × NS hybrids and analyzed their reprograming. The ESCs were BIO pretreated for different times, labeled with DiD, and then cocultured with NS-Oct4-puro cells labeled with DiO. The efficiency of fusion was comparable for each BIO treatment time point (up to 2% of total cells), as seen by FACS analysis (Fig. 3A). However, a high number of reprogramed clones (up to 25-fold reprograming) was selected only when we plated the DiD/DiO-positive sorted hybrids derived from the fusion of NSCs with 24-hour-BIO pretreated ESCs (Fig. 3B). This again confirms that spontaneous fusion can occur, although with low efficiency; however, the hybrids undergo reprograming and proliferation only after time-dependent activation of Wnt signaling.

To show that the hybrids that do not undergo reprograming after cell fusion embark into apoptosis, we analyzed the sorted ES × NS hybrids for their apoptotic phenotype. In all, 10,000 fused cells (DiD/DiO positive) were sorted for each of the time points. After the sorting, the hybrids were plated and harvested 48 hours later. Here, we observed that hybrids derived from 12-hour- and 48-hour-treated ESCs or from untreated ESCs cocultured with NSCs showed a greater apoptotic phenotype as compared with hybrids derived from the 24-hour-BIO-treated ESCs cocultured with NSCs (Fig. 3C), which is the point that shows more reprograming. As control, we also analyzed apoptosis of ES- or NS-sorted cells. As expected, sorted cell fusion hybrids showed a greater apoptotic phenotype when compared with single-sorted cells (Fig. 3C). This result clearly shows that hybrids heading into reprograming embark into apoptosis with low efficiency.

Next, to determine whether by blocking apoptosis, we can isolate a greater number of reprogramed clones, we used z-VAD, which is a general inhibitor of the activation of the apoptosis program [22,23]. Two increasing doses of z-VAD efficiently blocked citrinin-induced apoptosis in ESCs (Fig. 3D). Then, NS-Oct4-puro cells were cocultured with untreated ESCs or with 24-hour-BIO-treated ESCs, and the reprogramed hybrids were selected after being cultured with z-VAD for 36 hours. Interestingly, a slight increase in the number of reprogramed clones was seen from the untreated ES × NS hybrids when the apoptosis inhibitor was added. In contrast, as expected, the reprograming efficiency was comparable in the presence or absence of z-VAD after the coculture of 24-hour-BIO-treated ESCs with NSCs, as these hybrids do not enter into apoptosis (Fig. 3D). These data show that a block of the apoptosis program can increase the reprograming of NSCs after spontaneous fusion; however, the efficiency of this process is very poor if the Wnt/β-catenin signaling pathway is not previously activated in the ESCs.

### The Reprograming of Spontaneously Formed Hybrids is Dependent on β-Catenin Accumulation up to a Specific Threshold in ESCs

**Figure 4:**
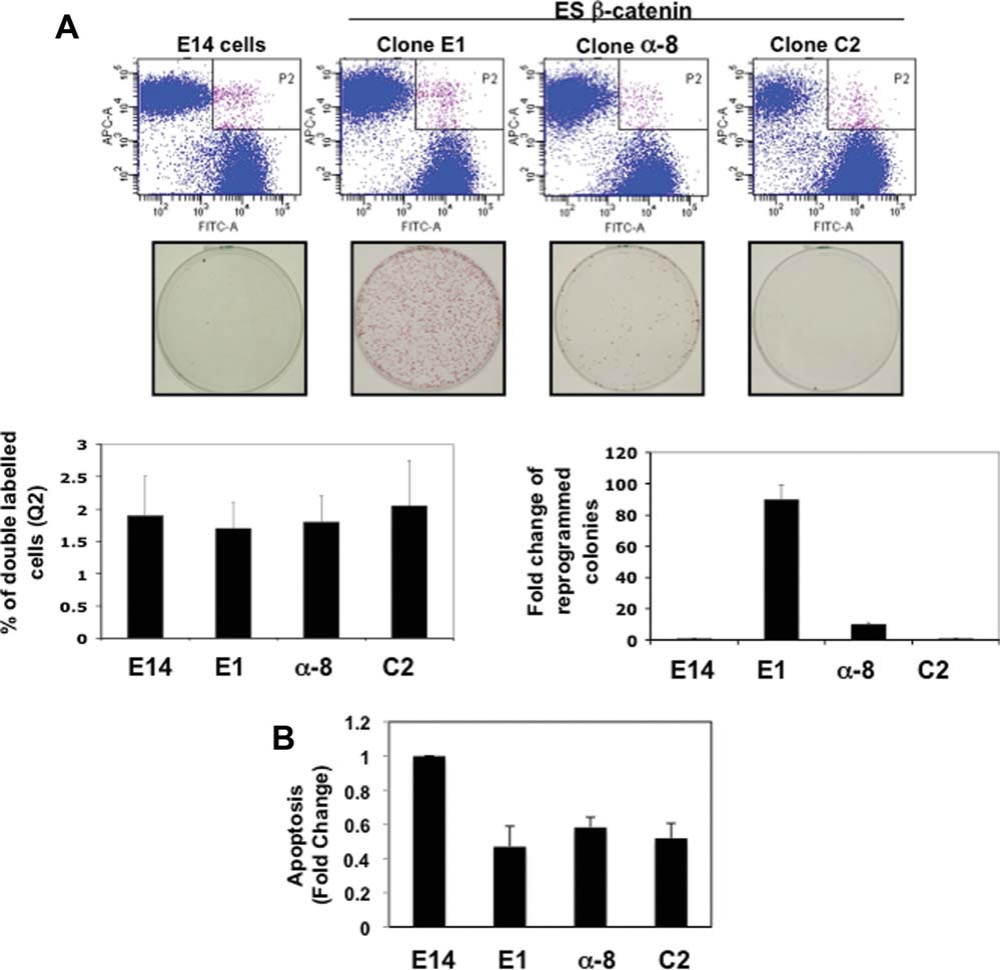
Low levels of stabilized β-catenin induce reprograming of NS cells through spontaneous cell fusion. **(A):** Quantification of FACS analysis (% total double-labeled cells) and representative growth plates with quantification of reprograming efficiency (fold-increase in colony number) of the cocultured cells (mean ± SEM; *n* = 3), for wild-type ESCs (E14), ES-β-catenin clones (E1, α-8, C2) with NS-Oct4-puro cells (mean ± SEM; *n* = 3). **(B):** 1 × 10^4^ sorted cell fusion hybrids between wild-type ESCs, ES-β-catenin clones (E1, α-8, C2) with NS-Oct4-puro cells were plated and apoptosis was evaluated using Cell Death Detection Elisa kits 48 hours after sorting (mean ± SEM; *n* = 3). Fold changes were calculated with respect to wild-type ESC × neural stem cell hybrids. Abbreviations: ES, embryonic stem; FACS, fluorescence activated cell sorting; FITC, fluorescein isothiocyanate.

To further demonstrate that a fixed level of β-catenin is important for the reprograming of somatic cells, the β-catenin-expressing ES clones were cocultured with NS-Oct4-puro cells. Before coculturing, ES-β-catenin clones were labeled with DiD and NSCs with DiO. Then 80% of the cells were analyzed by FACS and 20% were plated to select reprogramed clones with puromycin. ESCs expressing low (the E1 clone), intermediate (the α-8 clone), and high (the C2 clone) levels of β-catenin fused with comparable efficiency (about 2%); however, a high number of reprogramed, AP-positive cells (750 reprogramed clones on average) were generated from only the hybrids spontaneously formed between the E1 clone and NSCs (Fig. 4A). These results demonstrate that only ESCs expressing low amounts of β-catenin can reprogram hybrids that they have spontaneously formed with NSCs.

We then investigated whether hybrids formed between the ES-β-catenin clones and NSCs underwent apoptosis. Interestingly, the FACS-sorted E1 × NS hybrids showed much lower levels of apoptosis, with respect to the untreated ES × NS hybrids. This further confirms that these hybrids that undergo reprograming do not enter into apoptosis. However, we observed low levels of apoptosis even in the α-8 × NS and C2 × NS hybrids, which did not generate reprogramed colonies (Fig. 4B). This was probably due to the high level of *p16^Ink4a^* and *p19^Arf^* in these cells (Fig. 1F), which would force them to arrest in a G0-senescent state. These data further confirm that β-catenin does not retain an antiapoptotic effect, as hybrids formed between ESCs expressing high levels of β-catenin and NSCs did not enter into apoptosis.

c-Myc is a target of β-catenin [24], and it can enhance direct reprograming when overexpressed in somatic cells along with Oct4, Klf4, and Sox2 [25]. Furthermore, c-Myc controls the cell cycle and apoptosis [26–28]. Thus, c-Myc is a good candidate as a downstream target of β-catenin in the enhancement of reprograming. We previously demonstrated that *c-Myc* was not transcriptionally activated in ESCs treated for different times with BIO [5], and also others have excluded its role in direct reprograming experiments when MEFs were transduced with Oct4, Sox2, and Klf4 and cultured in the presence of Wnt3a [19]. However, to further confirm these results, we have analyzed the level of the c-Myc protein and of phosphorylated c-Myc in ESCs treated for 12–48 hours with BIO and in β-catenin clones. The levels of c-Myc and of phosphorylated c-Myc were comparable in untreated ESCs, in all of the treated ESCs, and in all of the β-catenin clones. An increase in total c-Myc and a decrease in phosphorylated c-Myc were instead seen in GSK3−/− ESCs, as expected [29] (supporting information Fig. S4). These data rule out a role for c-Myc as a β-catenin-dependent reprograming factor.

The reprogramed phenotype of the AP-positive and puromycin-resistant clones was confirmed by performing in vitro and in vivo differentiation studies. The clones were tetraploids (supporting information Fig. S5A) and expressed GFP even after several passages (supporting information Fig. S5B). In addition, they expressed stem cell markers, such as *Oct4*, *Nanog*, and *Rex1*, but silenced the neural-specific markers *Blp* and *Olig2* (supporting information Fig. S5C). CpG islands were demethylated in the promoter regions of *Oct4* and *Nanog* of the reprogramed clones, which showed a methylation profile similar to the ESCs (supporting information Fig. S5D). Embryoid bodies and beating cardiomyocytes were formed with similar timing and efficiency by both reprogramed clones and ESCs; likewise for the expression of markers for mesoderm (Brachyury), endoderm (AFP), and cardiac muscle (Nkx2.5) (supporting information Fig. S5E, F). We also observed that reprogramed clones differentiated into several tissue types in vivo (e.g., epidermis, gut-like epithelium, neural tissue, muscle, cartilage) on injection in the posterior legs of SCID mice, (supporting information Fig. S5G). All of these data demonstrate that the selected reprogramed clones were pluripotent, as they differentiated in vitro and in vivo.

## DISCUSSION

**Figure 5:**
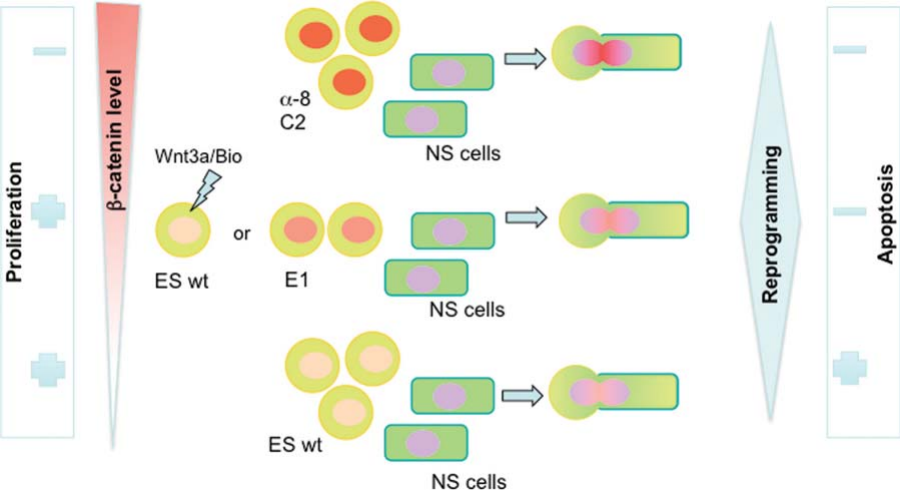
Spontaneous-fusion-mediated cell reprograming is controlled by β-catenin levels in ESCs and not by their proliferation. Wild-type ESCs and ES clones expressing low and high β-catenin levels fuse with Neural stem cells (NSCs) spontaneously with the same low efficiency. Only the hybrids formed by NSCs fused with Wnt3a-/BIO-treated ESCs or the E1 β-catenin clone become reprogramed and can be selected with high efficiency. In contrast, hybrids formed by NSCs fused with wild-type ESCs enter into apoptosis-mediated cell death. Interestingly, the hybrids formed by NSCs fused with α-8 and C2 clones, which express high levels of β-catenin, neither become reprogramed nor enter into apoptosis or proliferate. This is likely due to the high levels of p16^Ink4a^ and p19^Arf^, see main text for details. Interestingly, β-catenin levels in ESCs modulate their ability to proliferate; however, ESC proliferation does not modulate cell fusion-mediated reprograming efficiency. Abbreviations: BIO, 6-bromoindirubin-3′-oxime; ES, embryonic stem; NS, neural stem.

In this study, we have demonstrated that spontaneous fusion can occur simply by coculturing ESCs with NSCs. This is a very inefficient process in vitro. Indeed, we have shown that the spontaneously formed hybrids embark into apoptosis, and therefore cannot be selected. In contrast, when the Wnt signaling pathway is activated in ESCs and leads to a fixed amount of β-catenin accumulation in the nucleus, then the spontaneous hybrids can undergo reprograming and be selected with high efficiency. ESCs expressing whatever amount of β-catenin can fuse, but normally the fate of the resulting hybrids is to undergo apoptosis, unless low levels of nuclear β-catenin allow them to undergo reprograming instead (see scheme in Fig. 5). This is not merely due to a Wnt-mediated survival effect, indeed, β-catenin does not protect ESCs from apoptosis (Fig. 1), and inhibition of the apoptotic pathway does not greatly increase the efficiency of spontaneous fusion-mediated reprograming (Fig. 3D). These observations indicate that β-catenin increases the reprograming of the hybrid cells, and as a result, the hybrids do not enter into the apoptotic pathway.

Spontaneously formed hybrids enter into apoptosis to avoid catastrophic consequences. This might well be a safety control mechanism that blocks the occurrence of aneuploidy and of genomic instability that would lead to tumor development. Indeed, increased resistance to apoptosis of cancer stem cells has been associated with fusion events. Spontaneous stable hybrids formed after fusions of breast cancer stem cells with breast cancer cells showed increased expression of ATP-binding cassette (ABC) multidrug resistance transporters and of antiapoptotic molecules [30]. On the other hand, during normal tissue homeostasis, the apoptotic pathway might lead to removal of detrimental fusion events. Here, we show that the hybrids can also face a different developmental fate: reprograming and survival from the death mediated by apoptosis.

Wnt is an important pathway that can induce cell proliferation and tumorigenesis, which is mainly due to Wnt-dependent expression of c-myc and cyclin D1 [31]. We and others have shown that neither of the genes for c-myc or cyclin D1 are activated when β-catenin accumulates in ESC nuclei [5,32]. On the other hand, the proliferative state of the fusing ESCs might also have a function in modulating reprograming efficiency after spontaneous fusion. Here, we have shown that low levels of β-catenin (Wnt/BIO-treated cells or the E1 clone) in ESCs have no effect for increasing proliferation compared with untreated ESCs. However, if β-catenin levels are increased (as in the case of the α-8 and C2 clones), a strong reduction in cell proliferation is seen (Fig. 5). This dual effect of Wnt signaling in proliferation has already been described in other systems. Interestingly, one study revealed that low levels of Wnt signaling stimulate human mesenchymal stem cell proliferation, while high levels have an inhibitory effect [33]. Moreover, the overexpression of β-catenin in neuroblastoma cells has been shown to result in an important reduction in proliferation [34].

Interestingly, we found that the low-proliferating α-8 and C2 clones show a reduced ability to reprogram somatic cells, even if they can spontaneously fuse with the same efficiency as the E1 clone. This latter clone, in contrast, retained very high fusion-mediated reprograming activity (Fig. 5). This suggests that high levels of β-catenin can induce the expression of some genes that might block reprograming. Of note, we observed that α-8 and C2 clones express high levels of the cell cycle regulators *p16^Ink4a^* and *p19^Arf^*, which have already been shown to inhibit reprograming [35–40].

Thus, it appears that the levels of β-catenin can modulate proliferation, although this is not the main mechanism enhancing reprograming. Some β-catenin target genes are expressed only when the level of β-catenin reaches a specific threshold; these should be important factors in the reprograming of somatic cells.

On the other hand, the anti-proliferative effect due to the increased level of the tumor suppressor genes *p16^Ink4a^* and *p19^Arf^* can also have antiapoptotic functions. In fact, it is well know that oncogenes promoting cell cycle progression also sensitize cells to express proapoptotic factors [41]. For this reason, the hybrids formed by NSCs and α-8 and C2 clones did not embark into apoptosis even if they were not reprogramed.

Fusion of ESCs and somatic cells produces reprogramed hybrid cells when there is β-catenin accumulation or Nanog overexpression [5,21,42]. However, we showed previously that in cell fusion-mediated reprograming where both Nanog were overexpressed and Wnt signaling was activated, the reprograming of somatic cells was very high, but the two pathways remained distinct. Nanog-mediated reprograming was not dependent on activation of the Wnt/β-catenin pathway. Furthermore, Nanog was not a β-catenin-dependent target gene [42].

In conclusion, if ESCs fuse spontaneously, this might perhaps also hold true for adult stem cells that might fuse with somatic cells to eventually control differentiation of the fusing partners. Thus, cell-cell fusion also appears to represent a cell fate and differentiation mechanism that if deregulated, might become a mechanism for tumor development.

## DISCLOSURE OF POTENTIAL CONFLICTS OF INTEREST

The authors indicate no conflicts of interest.
