# Supplementary figures and images for: The Wnt/β-Catenin Signaling Pathway Tips the Balance Between Apoptosis and Reprograming of Cell Fusion Hybrids
Source: Stem Cells. 2010 Nov;28(11):1940–9. doi: 10.1002/stem.515 (PMC3003905; doi:10.1002/stem.515)

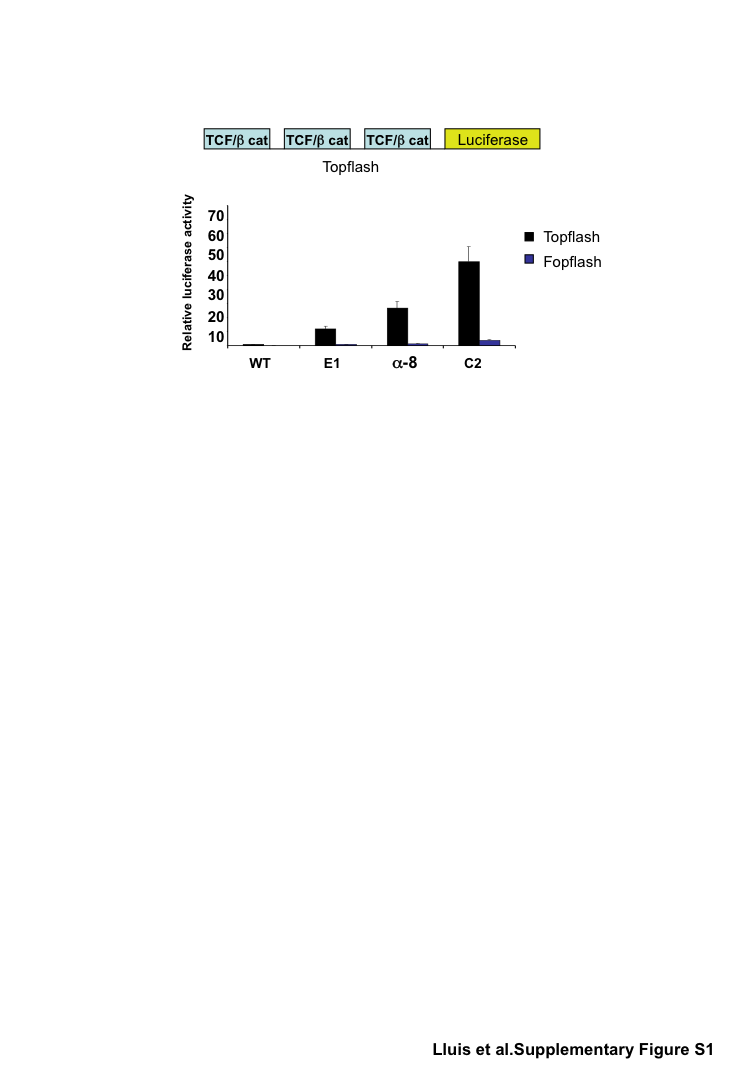

Supplement: Supplementary file 1 [file stem0028-1940-SD1.tif]

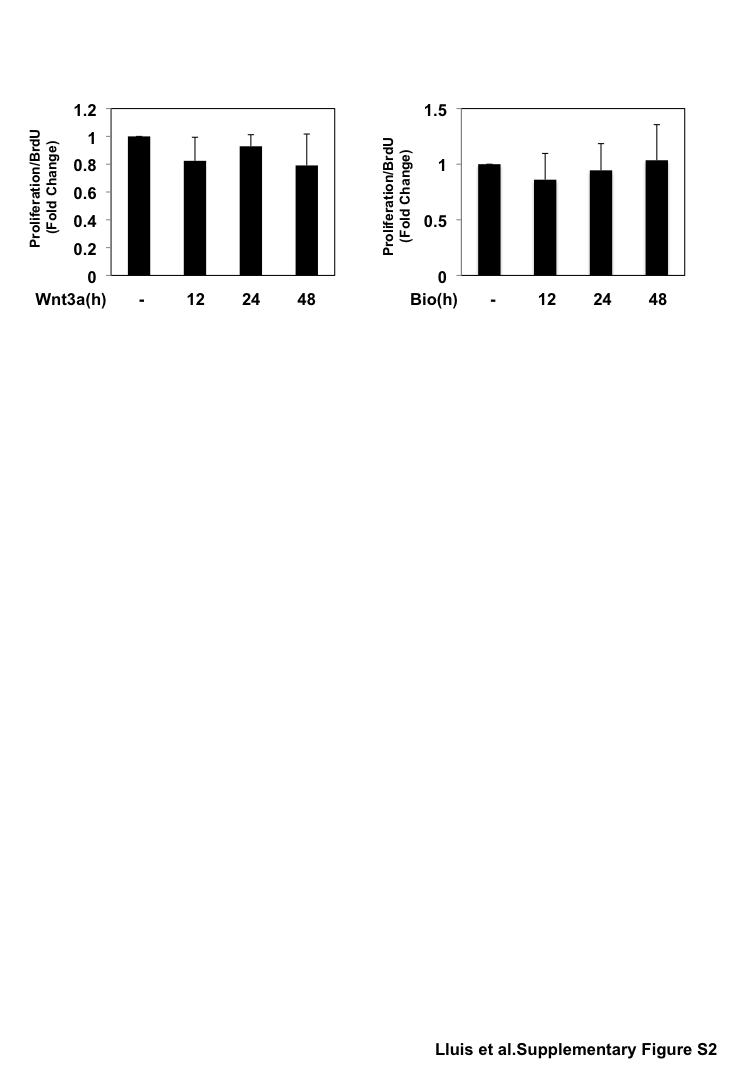

Supplement: Supplementary file 2 [file stem0028-1940-SD2.tif]

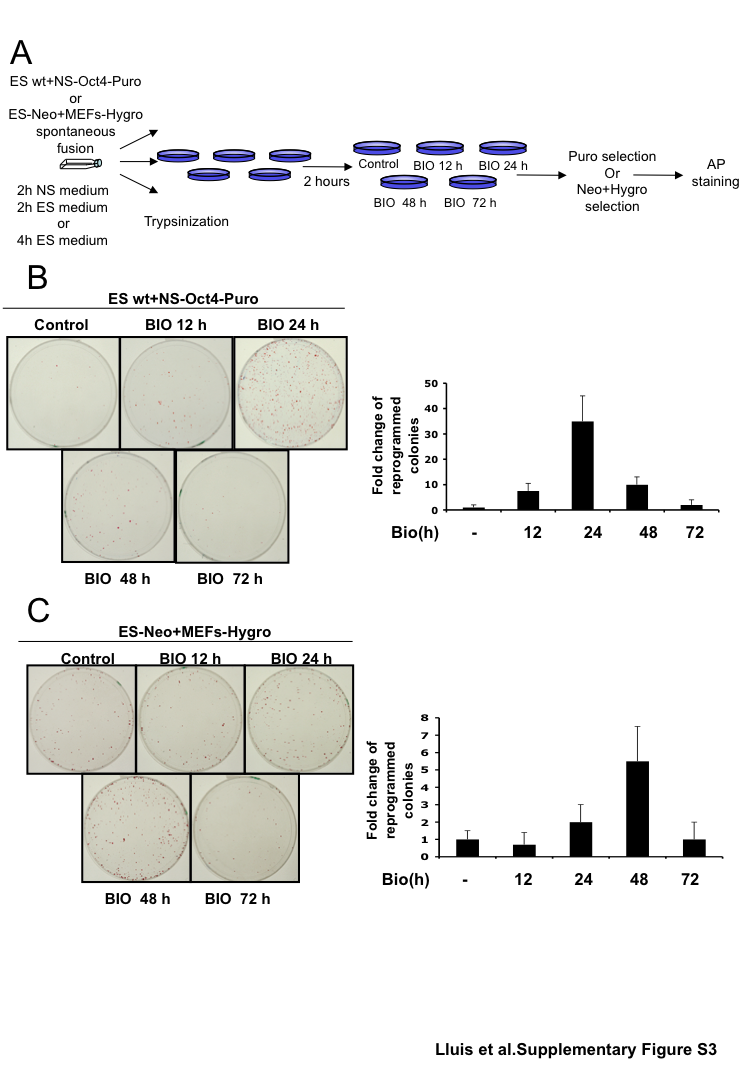

Supplement: Supplementary file 3 [file stem0028-1940-SD3.tif]

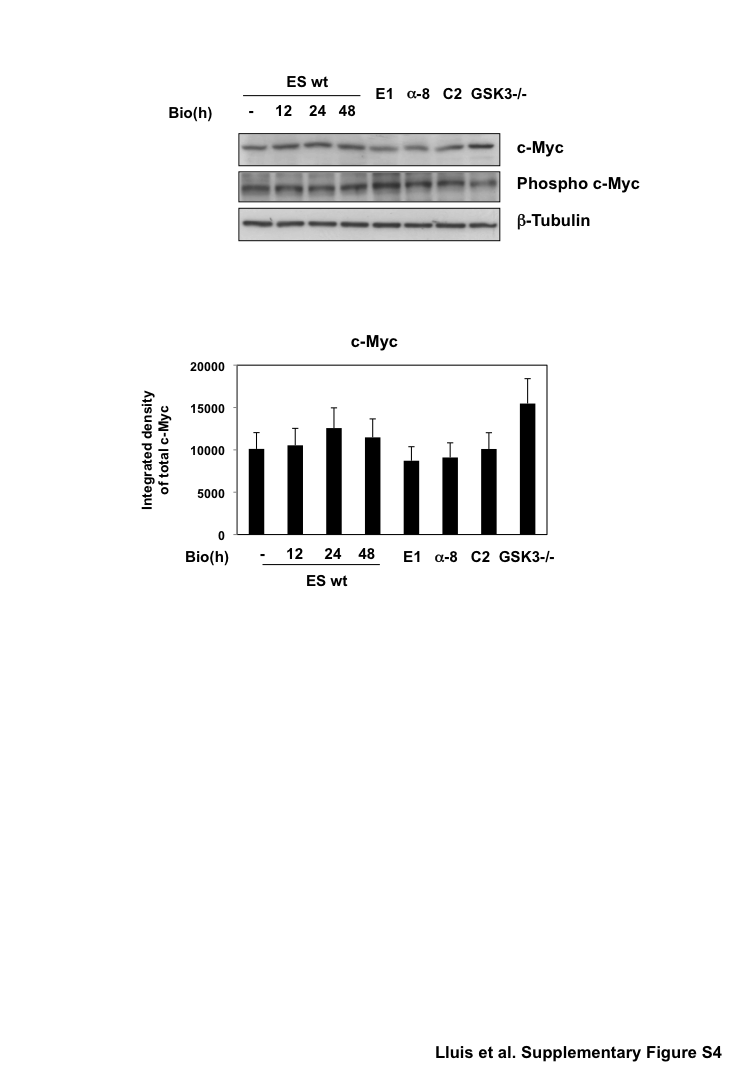

Supplement: Supplementary file 4 [file stem0028-1940-SD4.tif]

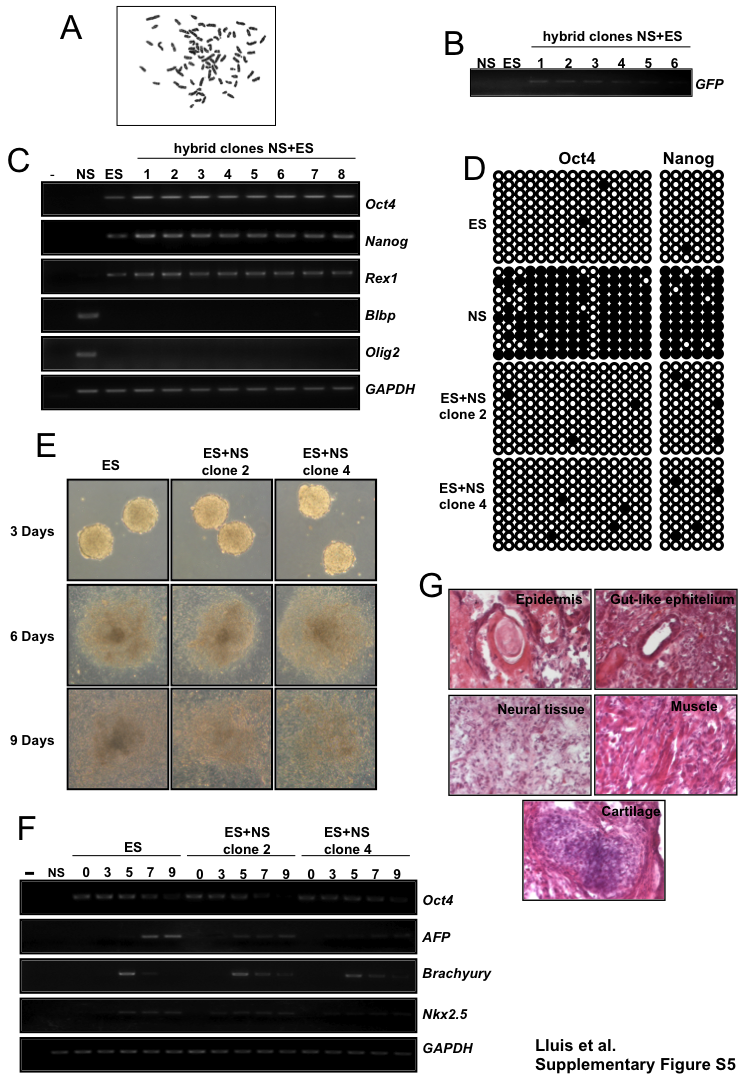

Supplement: Supplementary file 5 [file stem0028-1940-SD5.tif]
